# Beware the Unpredictability of Modified UNICORN

**DOI:** 10.1016/j.jaccas.2025.106205

**Published:** 2026-03-18

**Authors:** Toby Rogers, Vasilis C. Babaliaros, Robert J. Lederman, Jaffar M. Khan, Adam B. Greenbaum

**Affiliations:** a MedStar Washington Hospital Center, Washington, DC, USA; b National Heart, Lung and Blood Institute, National Institutes of Health, Bethesda, Maryland, USA; c Structural Heart and Valve Center, Emory University Hospital, Atlanta, Georgia, USA; d St Francis Hospital and Heart Center, Roslyn, New York, USA

Aortic valve leaflet modification has evolved from BASILICA to include a wide range of transcatheter techniques to lacerate, disrupt, or even remove leaflets to prevent coronary obstruction during transcatheter aortic valve replacement (TAVR).

The safety and effectiveness of BASILICA were demonstrated through careful preclinical, prospective multicenter studies and larger retrospective registries.^1^ Many of the more recent leaflet-modification techniques have not been subjected to similar scientific rigor.

UNICORN (or CLEVE) involves intraleaflet deployment of a balloon-expandable valve. Key procedure steps involve electrosurgical target leaflet traversal—like BASILICA. Then a balloon is inflated to create an orifice but not to completely tear the leaflet. Then a balloon-expandable valve is deployed within the leaflet. On the benchtop, valve deployment effectively avulses the leaflet, trapping material on the contralateral side.^2^ However, anecdotally, it can be challenging to deliver the transcatheter heart valve into the leaflet orifice.

In a recent issue of *JACC: Case Reports*, Giustino et al^3^ described a “modified UNICORN” technique in which a larger balloon is used to tear the leaflet before TAVR is performed. The key “modification” is the use of a larger balloon to complete the tear of the leaflet—rather than create an orifice for intraleaflet valve deployment. This approach may be simpler but creates great unpredictability. Tearing the leaflet to the free edge was never the intent of UNICORN.

First, inflating a large balloon creates uncontrolled laceration of the leaflet. Unlike BASILICA which creates a predictable linear laceration from leaflet base to tip, this modified UNICORN potentially creates a jagged laceration around calcification, tears the leaflet off a commissure, or avulses the leaflet altogether. The resulting flail leaflet risks coronary obstruction or embolization.

Second, this approach risks hemodynamic collapse from torrential aortic regurgitation. Anecdotally, we are aware of cases of emergency valve deployment during pulseless electrical activity arrest caused by leaflet avulsion. In BASILICA, degenerated leaflets typically co-apt adequately after linear laceration avoiding severe regurgitation. In contrast, this modified UNICORN destroys the target leaflet. The authors caution need for haste in deploying the valve. But it is undesirable to create a situation in which the TAVR must be performed hurriedly under unstable hemodynamic conditions.

Operators should exercise caution before adopting leaflet-modification techniques which have not been rigorously tested and risk serious complications.
